# Supplementary material for: Using aquatic animals as partners to increase yield and maintain soil nitrogen in the paddy ecosystems
Source: eLife. 2022 Feb 22;11:e73869. doi: 10.7554/eLife.73869 (PMC8863371; doi:10.7554/eLife.73869)
Supplement: Transparent reporting form [file elife-73869-transrepform1.docx]

***eLife’s* transparent reporting form**

We encourage authors to provide detailed information *within their submission* to facilitate the interpretation and replication of experiments. Authors can upload supporting documentation to indicate the use of appropriate reporting guidelines for health-related research (see [EQUATOR Network](http://www.equator-network.org/%20)), life science research (see the [BioSharing Information Resource](https://biosharing.org/)), or the [ARRIVE guidelines](http://www.plosbiology.org/article/info:doi/10.1371/journal.pbio.1000412) for reporting work involving animal research. Where applicable, authors should refer to any relevant reporting standards documents in this form.

If you have any questions, please consult our Journal Policies and/or contact us: [editorial@elifesciences.org](mailto:editorial@elifesciences.org).

**Sample-size estimation**

- You should state whether an appropriate sample size was computed when the study was being designed
- You should state the statistical method of sample size computation and any required assumptions
- If no explicit power analysis was used, you should describe how you decided what sample (replicate) size (number) to use

Please outline where this information can be found within the submission (e.g., sections or figure legends), or explain why this information doesn’t apply to your submission:

1) We did three field experiments with completely randomized block design and six replicates. The information was provided in “Materials and methods” section in the main text, and in the section “Appendix 4 Field experiment details”.

2) For testing organic matter decomposition rate, we randomly buried 20 tubes in each plot, and retrieved five tubes from each plot. The information was provided in the section “Appendix 5 Method for testing organic matter decomposition rate”.

3) We did three mesocosm experiments with completely randomized block design, with six replicate blocks. The information was provided in “Materials and methods” section in the main text.

**Replicates**

- You should report how often each experiment was performed
- You should include a definition of biological versus technical replication
- The data obtained should be provided and sufficient information should be provided to indicate the number of independent biological and/or technical replicates
- If you encountered any outliers, you should describe how these were handled
- Criteria for exclusion/inclusion of data should be clearly stated
- High-throughput sequence data should be uploaded before submission, with a private link for reviewers provided (these are available from both GEO and ArrayExpress)

Please outline where this information can be found within the submission (e.g., sections or figure legends), or explain why this information doesn’t apply to your submission:

1) Each of three field experiments (one each for rice-carp, rice-crab, and rice-turtle system) was conducted for 4 years (2017-2020) at a site (one system per site). In each experimental year (from 2017 to 2020), rice grain and fresh aquatic animals were harvested from the whole experimental plots. The information was provided in the “Materials and methods” section in the main text.

2) In 2018, the stable-isotope ^13^C labeling method was used to determine the organic matter decomposition rate in the three field experiments. The information was provided in the “Materials and methods” section in the main text.

3) During the rice growing period in 2019, we collected living organisms that were consumed by the aquatic animals in each of the three experiments. The information was provided in the “Materials and methods” section in the main text.

4) We conducted three independent mesocosm experiments (rice-carp, rice-crab, or rice-turtle systems) at the Experimental Station of Zhejiang University in Deqing County, Zhejiang Province in 2019. The information was provided in the “Materials and methods” section in the main text.

5) All our field experiments have six block replications, and we include all result data without exclusion.

**Statistical reporting**

- Statistical analysis methods should be described and justified
- Raw data should be presented in figures whenever informative to do so (typically when N per group is less than 10)
- For each experiment, you should identify the statistical tests used, exact values of N, definitions of center, methods of multiple test correction, and dispersion and precision measures (e.g., mean, median, SD, SEM, confidence intervals; and, for the major substantive results, a measure of effect size (e.g., Pearson's r, Cohen's d)
- Report exact p-values wherever possible alongside the summary statistics and 95% confidence intervals. These should be reported for all key questions and not only when the p-value is less than 0.05.

1) All data were subjected to a homogeneity test. If the data did not meet the assumptions of normality and homogeneity, they were log-transformed before analysis. The information was provided in the “Materials and methods” section in the main text.

2) Statistical analysis was conducted by using the GLM in SPSS (V.20.0). For each field experiment, ANOVAs with a split-plot design (i.e., treatment as the main plot, and experimental year as the sub-plot) were performed on rice yields, total soil N content, ANUE, and weed biomass. The information was provided in the “Materials and methods” section in the main text.

3) For RM or RA mesocosms, δ^15^N in the soil at the beginning and end of the experiment was compared by using paired t-tests (SPSS 461 V.20.0). This information was provided in the “Materials and methods” in the main text.

4) We compare all the SM and SA results by F-test, but compare δ^15^N at the beginning with the end by t-test. The values of each treatment presented in all pictures are means±SE, and we provide exact p-values for all comparison no matter the result is significant or non-significant.

Please outline where this information can be found within the submission (e.g., sections or figure legends), or explain why this information doesn’t apply to your submission:

(For large datasets, or papers with a very large number of statistical tests, you may upload a single table file with tests, Ns, etc., with reference to sections in the manuscript.)

**Group allocation**

- Indicate how samples were allocated into experimental groups (in the case of clinical studies, please specify allocation to treatment method); if randomization was used, please also state if restricted randomization was applied
- Indicate if masking was used during group allocation, data collection and/or data analysis

Please outline where this information can be found within the submission (e.g., sections or figure legends), or explain why this information doesn’t apply to your submission:

1) We did three field experiments with completely randomized block design, treatments were randomly assigned to each of six blocks of one experiment, the information was provided in “Materials and methods” section in the main text, and in the section “Appendix4 Field experiment details”.

2) We did three mesocosm experiments with completely randomized block design, with six replicate blocks, treatments were randomly assigned to each of six blocks of one experiment. The information was provided in “Materials and methods” section in the main text.

**Additional data files (“source data”)**

- We encourage you to upload relevant additional data files, such as numerical data that are represented as a graph in a figure, or as a summary table
- Where provided, these should be in the most useful format, and they can be uploaded as “Source data” files linked to a main figure or table
- Include model definition files including the full list of parameters used
- Include code used for data analysis (e.g., R, MatLab)
- Avoid stating that data files are “available upon request”

Please indicate the figures or tables for which source data files have been provided:

1) All data generated or analyzed during this study are included in the manuscript and supporting file1-7. Source Data files 1-6 and source Data files 7-9 and have been provided for Figures 1-6 and Appendix 1-figure 1-3, respectively.

2)Dietary contributions of input feed and potential food sources from the rice field were analyzed by stable isotopic dietary reconstruction with the R package “siar”, the information was provided in “Materials and methods” section in the main text.
